# Supplementary figures and images for: Testing the oceanic dispersal potential of Caribbean fleshy-fruited plants
Source: PLoS One. 2026 May 27;21(5):e0348628. doi: 10.1371/journal.pone.0348628 (PMC13215481; doi:10.1371/journal.pone.0348628)

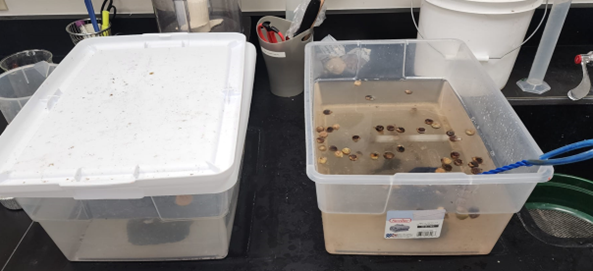

Supplement: S1 Fig — (PNG) [file pone.0348628.s001.png]

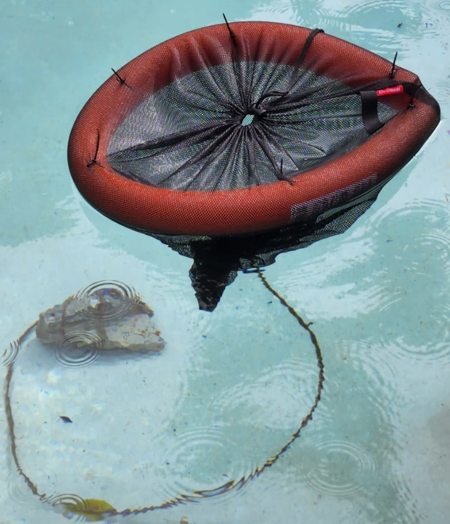

Supplement: S2 Fig — (PNG) [file pone.0348628.s002.png]

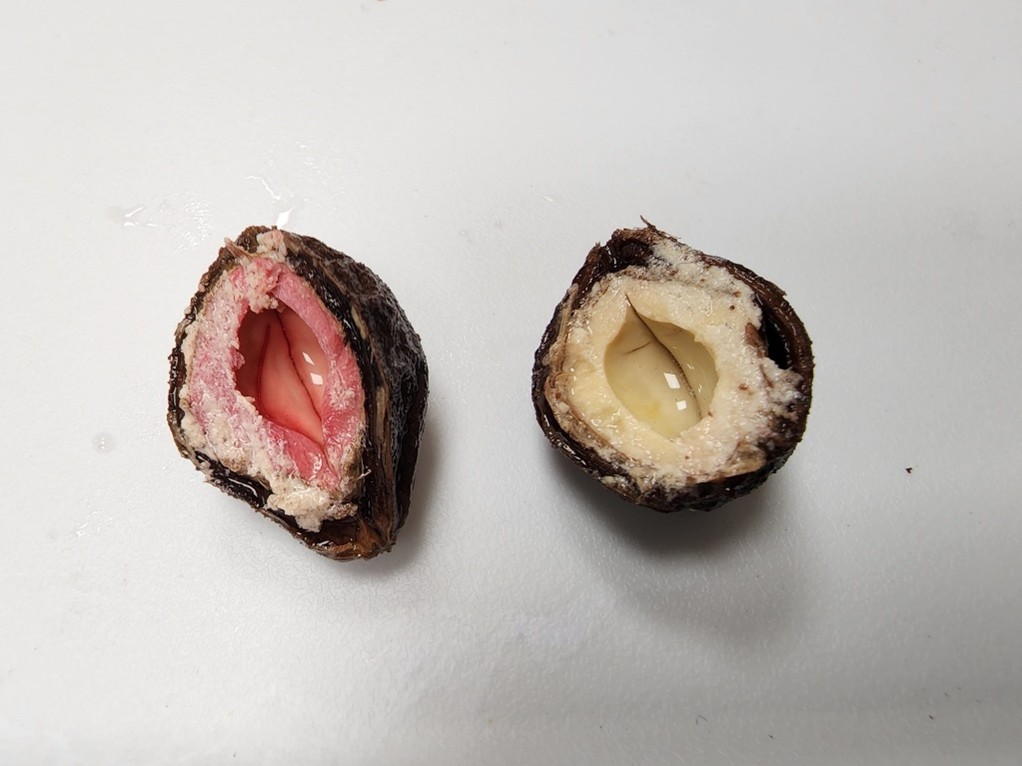

Supplement: S3 Fig — Viable seeds are stained red (left), while non-viable seeds are not (right). (JPG) [file pone.0348628.s003.jpg]

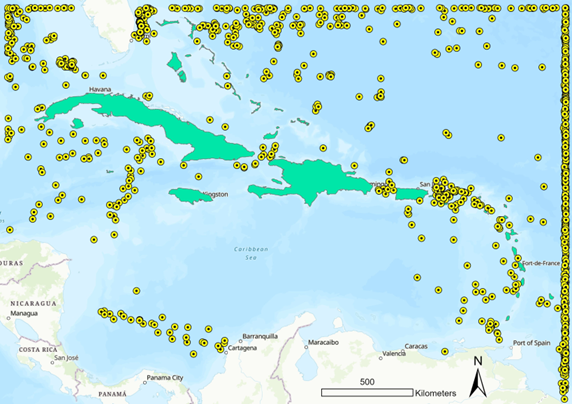

Supplement: S4 Fig — Yellow circles are starting points for all of our buoys. (PNG) [file pone.0348628.s004.png]

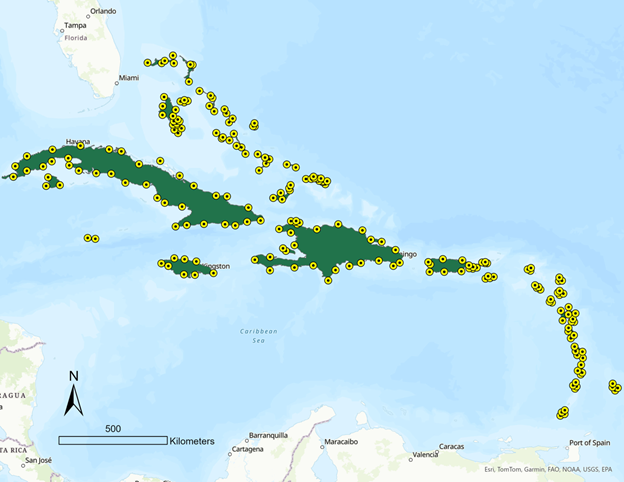

Supplement: S5 Fig — (PNG) [file pone.0348628.s005.png]

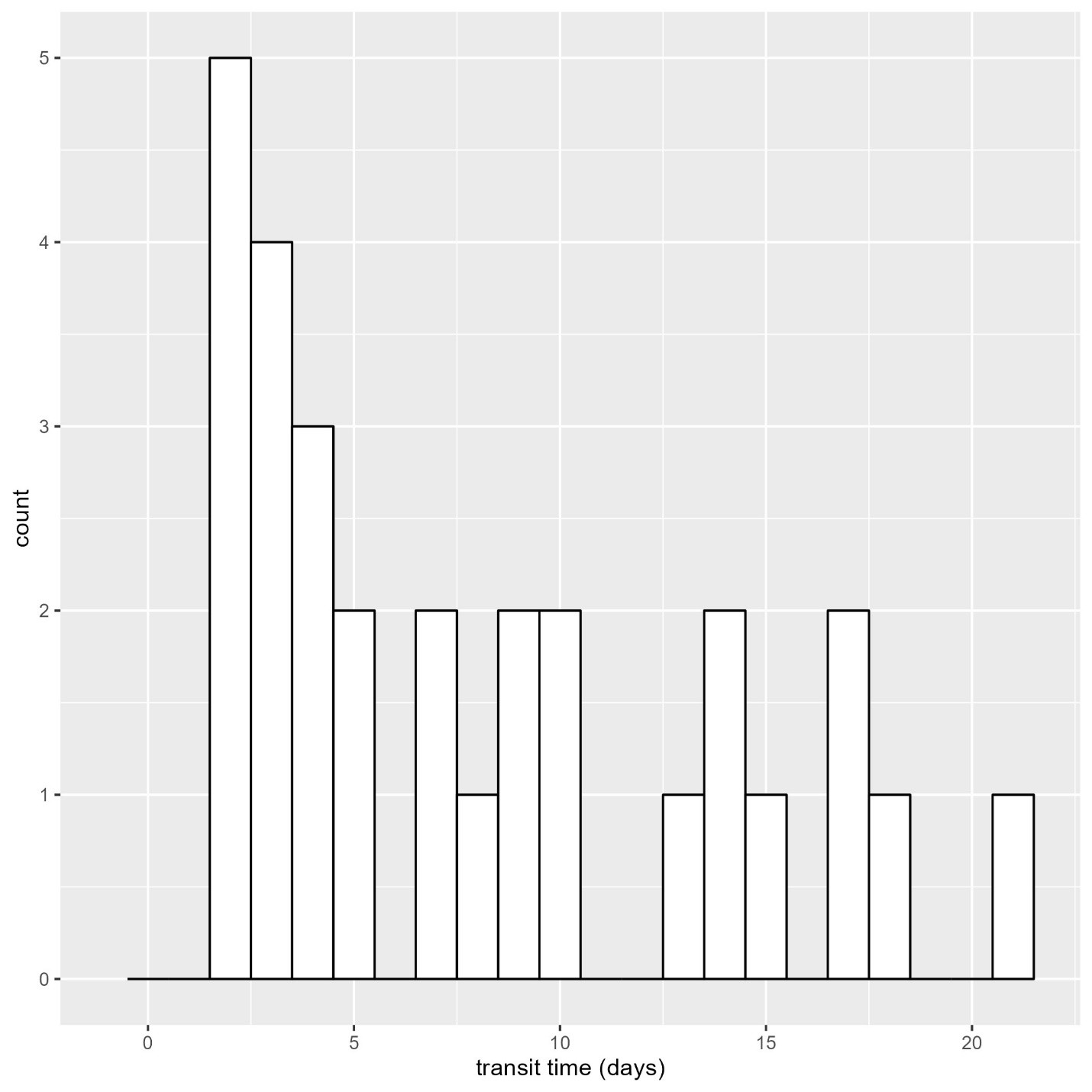

Supplement: S6 Fig — For all distributions, see Github link. (JPG) [file pone.0348628.s006.jpg]
