# Supplementary material for: Testing the oceanic dispersal potential of Caribbean fleshy-fruited plants
Source: PLoS One. 2026 May 27;21(5):e0348628. doi: 10.1371/journal.pone.0348628 (PMC13215481; doi:10.1371/journal.pone.0348628)
Supplement: Attachment — Submitted filename: Response to Reviewers.docx [file pone.0348628.s014.docx]

Seokmin Kim

Reviewer Responses

PLOS One

October 29, 2025

We are ready to submit a revised version of the manuscript now titled: “Testing the potential of Caribbean fleshy-fruited plants to disperse using ocean currents”. We deeply appreciate everyone’s efforts in providing such thorough comments and suggestions, and we believe that incorporating everyone’s constructive feedback has been very helpful in making the manuscript stronger. With our narrative, we followed Reviewer 2’s comments and restructured our introduction to focus more on the potential of thalassochory (ocean current mediated dispersal) to be an alternative form of dispersal for fleshy-fruited plants, which primarily utilize endozoochory. With this, we aimed to dampen our previous suggestion that thalassochory may replace animal dispersers and instead stressed how we are examining an alternative, lesser studied dispersal method for these fleshy-fruited plants. We also modified our statistical analysis and found some significant trends that we had overlooked previously. These changes are in addition to the numerous smaller changes that we have made throughout the manuscript.

Please see below for our responses to each reviewer. Our responses are in red.

Also, regarding your concern regarding copyright issues on Figure 1: We made the background continental and island layers ourselves, therefore we have no copyright information to declare. Please let us know if you need more information.

Reviewer #1: I enjoyed reading the manuscript by Kim et al. exploring the thalassochoric potential of Caribbean fleshy fruits and their correlates with distribution range, island distance and estimates of oceanic connectivity. The paper explored a clearly understudied issue and contains a lot of high-quality data (I particularly liked the ocean connectivity analysis based on tagged buoys from NOAA) and solid analysis.
I have several important concerns about the framework in which some results are presented and discussed but I hope most can be solved with an in-depth revision. I also list a large number of minor corrections and suggestions that I hope can further assist the authors in improving their valuable work.

General points
I feel that the paper mixes a bit the role of thalassochory for colonisation of new islands (natural range expansions) and the role of dispersal for maintain gene flow and viable populations. For example, thalassochory is argued to be particularly important in the context of current defaunation. This rational isn’t completely wrong, but I think that there should be a better separation between the conservation and the biogeography issues, as the latter occurs on a much slower time scale. For reference, natural colonization’s of new species arriving to an island (those that can increase range) is a process that occurs on average only once every several thousand years (so this issue is largely decoupled from the issue of defaunation/conservation).

Thank you for your helpful feedback on this manuscript. We agree that thalassochory is important for colonization of islands. However, as a mechanism for Long Distance Dispersal (LDD), we believe that it still has a role in shaping gene flow. In our revised manuscript, we focus more on this aspect than on how thalassochory could potentially replace animal dispersers altogether. Additionally, we stress how we are examining a potentially secondary, abiotic alternative dispersal mechanism of fleshy-fruited plants (whose primary dispersal mechanism is endozoochory).

I’m puzzled (probably like the authors) that there is no effect between fruit density and flotation capacity. Doesn’t that go against the laws of physics? I guess that objects should float on liquids only if they have a lower density than the liquid. So, density is by definition related to flotation potential. There is some suggestion that water absorption could disrupt flotation (L375), but I’m not sure whether this can be the case as water cannot make any object denser than water itself. Maybe I’m not getting my physics right (sorry if that’s the case), but I think this issue deserves a more solid explanation. Actually, it would also be positive to explain beforehand why this is an important research question (why isn’t just a straightforward physical definition that all fruits denser than water will sink). Maybe impulsion, salinity and superficial tension forces need to be invoked here.

Regarding the discrepancy between fruit density and flotation capacity: we expanded on our manuscript to address this. See lines 390 to 394 (“Given that density did not predict floating potential of fruits, we suggest that water permeability may be one trait that predicts how well fruits can float. Fleshy-fruited plants are known to possess different levels of water permeability [61], and if water can penetrate a fruit’s exocarp and inundate the mesocarp, it could weigh down the fruit by displacing any air pockets and sink it, thus limiting its thalassochoric dispersal potential regardless of its seed traits.”). This is a similar principle as to why bread initially floats but then sinks as it becomes saturated with water.

The median 95th percentile viability time was used to classify species as “high” (>7 days) or low (<7 days) thalassochorous potential, but it wasn’t completely clear to me what this number actually means. Is this the median of all values within the 95th percentile (i.e. an estimate of a central measure of flotation) or is it the median of the values that represents the max. 95th percentile (i.e. an estimate of an extreme/near-maximum flotation capacity?). I think it makes more sense if it is a central measure, but please just clarify/confirm.

95^th^ percentile viability refers to when 95% of all fruits of a species had sunk / shown to be non-viable. Those values are in the 95^th^ percentile viable day column. The median value of that column is the metric we used to divide the study species between good and poor thalassochoric dispersers.

The relationship between species range (#islands) and viability while floating is only very very marginally significant at α=0.1 (p = 0.096) and most readers will probably consider this a non-significant result. This “extreme marginality” should be more clearly stated in the abstract and discussion. However, I wander (hope) if the relationship will be better with other measures of viability estimation (see previous comment/doubt about the median). Also, please add the result of the test directly into Fig. 3 to make life easier for the reader.
The abstract doesn’t say anything about viability and says very little about the current’s connectivity estimations (which are two important parts of the manuscript). I suggest the authors find space in the abstract to mention briefly these two points.

At the suggestion of another reviewer, we conducted a separated analysis (GLM with a random effect to account for taxonomy and with a Poisson error distribution) and found a significant relationship between species range size (number of islands) and species viability metrics (maximum / 95^th^ percentile) as predictor variables. We also rewrote the Abstract to better highlight the topics that the reviewer rightly identified as major outcomes of the study.

The fact that some islands have been connected by land bridges is only passively presented in the discussion, but it forms a strong constraint (and explanation) to the lack of an association between thalassochory connectivity and island distance. I feel this caveat should be assumed more clearly much earlier and probably it jeopardizes some of the analyses as it really doesn’t make much sense to assume the relevance of thalassochory for explaining the presence of a plant in two islands that have been connected for thousands of years in the past.

There is still much debate on the possibility of such a land bridge, which I found through additional literature review. Regardless of the existence of such a geologic feature, as such a bridge may have existed as far as 35 million years ago, I believe that over-water dispersal mechanisms may be more important for shaping current plant distributions. I elaborate more on this on lines 413 to 420.

“Additionally, current plant distributions may be relics of historic dispersal and connectivity patterns. The Caribbean archipelagos possess diverse geological histories, with some islands potentially having been connected to either the mainland or to other islands due to changing sea levels and tectonic movements [68]. However, there is still debate regarding this topic, particularly that of the hypothesized land-bridge between the Greater Antilles and South America (GAARlandia) approximately 34 million years ago [69, 70]. Regardless, while such geological histories could influence current plant distributions, the time scale of such geologic events suggest that over-water dispersal events could be more influential.”

Maybe I missed something, but I couldn’t find the supplementary tables mentioned in the Supplemental materials word document.

Apologies for that. The supplementary tables have been added.

Specific issues:
L35 its probably easier and shorter to say simply: 2600 km, rather than 2.6×10^3 km

Changed

L36, L98 “floating better” sounds a bit weird to me has it could be understand has having a lower fraction of the seed inside the water. Probably better replacing “better” by something like: longer or greater floating capacity.

Changed

L52 [3. 4]. -> [3, 4].

Changed

L56 “plays a potentially interesting role” not great choice of words. Maybe better “important” or “relevant”

Changed to “is particularly relevant”

L65 I’m not familiar with the term “turbulent dispersal”, maybe clarify.

Removed

L67 The study of Cryptocarpus pyriformis is also in the Caribbean? If not, consider mentioning the Archipelago

Added Galápagos Islands.

L77 has evolved traits facilitating this process -> has evolved traits to facilitate this process

Changed

L79 more large -> larger

Changed

L113 Figure 1 should be cited here

We cite Figure 1 just a few sentences later when we are done explaining everything that Figure 1 aims to show.

L117 [32 33]) -> [32, 33]).

Fixed

L125 consider: utilize -> benefit from

Changed to: “are most likely to benefit from”

Table 1 add a “.” After “diam” (diam.) and “max” (max.)

Added

L138 It sounds more logical to cite the Supplementary figures as: (Fig. S1) instead of (S1 Fig), but this might be a journal style, so I wont comment on that further.

This was a journal style.

L145 “free-moving/flowing salt water”. Not very clear, was this a bay? Or a swimming pool filled with saltwater and a circulation pump? Please clarify.

Clarified to “mangrove forest edge located within Montgomery Botanical Center”

See lines 153- 156: “In the second method, we placed fruits from each species within a body of free moving salt water (mangrove forest edge located within Montgomery Botanical Center). In addition to providing circulating salt water, this location also gave protection (through the presence of mangroves) from any major wave action that may have disturbed our study.**”**

L156 So this is a paired t-test for the differences for the 5 species used on both methods? Or a t-test for different species used in each method? Please add a bit more detail so that the reader can understand what was done. If there is no space in the main manuscript, this could be a small Supplement.

This was a paired t-test. Added that information to the main text. See line 166-168: “As we did not detect a difference in viability between the two methods through a paired t-test (t = 0.05, df = 4, p = 0.96), we combined our data from both methods for our analyses.”

L159 [39-40] -> [39, 40]

Changed

L199 The word “arbitrarily” seems a bit excessive here. Consider instead something like: “…44 major Caribbean islands (here considered as those larger than 100 km^2) “.

Changed

L236 (Fig 2) -> (Fig. 2) (find and replace throughout manuscript Fig -> Fig.)

This was a journal style.

L176, L243 Uniformize the way packages are written (with/without quotation marks; with/without italics)

Changed

L270, L283 Consider: there is significant variation in thalassochoric… -> …there are significant differences in thalassochoric

Changed

L271 You can abbreviate genus names after first mention.

Changed

L279 Consider: with 33 islands -> present in 33 out of 44 islands …

Changed

L279 indicate table with these results

Done

L300-305 This section could be written with a lot less words (avoiding some repetitions).

Made the section more concise. See line 317-320: “When comparing ocean-current–based dispersal metrics, both minimum dispersal time (ρ = 0.89, p < 0.001) and dispersal probability (ρ = –0.35, p < 0.001) were significantly correlated with geographic distance, but minimum dispersal time showed a stronger correlation (z = 18.31, p < 0.001).”

L311 “(insert stats).” Good idea :)

We have updated this analysis based on advice from another reviewer and now provide the statistics associated with these new findings. See lines 324-326: “This was accompanied by a significant, positive relationship between species range size and the sum of human use categories as well (Fig 3: max: z = 2.72, p = 0.006; 95th percentile: z = 3.36, p < 0.001). “

L311-319 This section is hard to read and carried a few weird and not very fluent text. Please try to improve readability.

Changed. See line 317-320. “When comparing ocean-current–based dispersal metrics, both minimum dispersal time (ρ = 0.89, p < 0.001) and dispersal probability (ρ = –0.35, p < 0.001) were significantly correlated with geographic distance, but minimum dispersal time showed a stronger correlation (z = 18.31, p < 0.001).”

L332 Best to highlight here that this relationship is very poorly supported (P=0.096)

We conducted a new analysis that now shows a significant relationship.

L410 Could this also be tested by contrasting the presence of plants in inhabited and uninhabited islands?

This is a very interesting suggestion, but as we were looking at the largest islands in the Caribbean, all of the islands in our study were inhabited. It would probably be challenging to get accurate occurrence records for smaller, uninhabited islands if relying on online databases such as we did here.

Reviewer #2: The manuscript entitled "Ocean current dispersal could benefit fruited plants in the Caribbean" by Seokmin Kim et al. evaluates the ability of 14 plant species native to the Caribbean islands to float and survive on salt water as a baseline to assess their potential for dispersal by ocean currents. The ability of ocean currents to shape and predict the distribution of these species is also assessed. The results indicate that these species, which are not a priori specialised for sea dispersal, differ in their ability to float and survive, and that this ability is directly related to the number of islands on which these species occur. However, no relationship was found between ocean currents dispersal metrics and species distribution. Assessing population connectivity and species dispersal ability is important to understand the degree of species isolation, particularly in restricted and isolated territories such as islands, in order to take effective conservation action and prioritize efforts and resources.

This study nicely combines an experimental approach and analytical techniques to assess the potential of species to be dispersed by ocean currents. The authors assess the floatability and viability of fruits in natural conditions (using an ingenious device), which is a valuable information to accurately evaluate whether a species is able to float and survive the minimum time necessary to be transported from one island to another. For this reason, I believe this manuscript has great potential to be published. However, I have two main concerns. My first concern has to do with the way this study has been framed. The interesting point about assessing thalassochory in these 14 species is that these species have fruit specializations that attract animals as dispersal vectors, and for which the sea was not thought to be the main dispersal agent. This decoupling between fruit specialization and long-distance dispersal has been extensively discussed in the literature (e.g. Heleno & Vargas, 2015; Higgins et al., 2003; Nathan, 2006), and the manuscript will be enriched if it is framed in this context. Following this suggestion will mean restructuring the introduction (see my suggestion below) and upgrading the discussion by including a broader topic of global interest. In addition, the bibliography cited in the discussion is rather poor. Regarding the role of ocean currents as dispersal vector, the authors can greatly improve the discussion of their results in light of previous studies that show their importance in transporting non-specialized organisms (e.g. lizards, Calsbeek & Smith, 2003), as well as specialized organisms (e.g. mangroves, Hodel et al., 2018; Ipomoea pes-caprae, Miryeganeh et al., 2014), studies conducted in the Caribbean region and in other parts of the world.

Thank you for your helpful feedback. We agree with your suggestion. We have now restructured our Introduction to better match your suggestion. We also incorporated a number of your suggested citations.

My second concern relates to the statistical analyses. The text is somewhat difficult to follow, and the use of some terms adds confusion (see my comments below). The authors rely mainly on Mantel tests, whose statistical power has been questioned. I believe that other models would allow the authors to test different hypotheses simultaneously (e.g. the role of sea dispersal, geography and human use/impact) and to draw stronger conclusions (see my suggestions below).

We have now supplemented our mantel tests with the GLM with poisson error distribution as you suggested below. This showed a significant relationship between species range size and viability potential (as well as human usage). We also attempted GDMs, but we could not get this to work properly. The package we tried using (“gdm”) struggled when not using coordinates (which would not be proper to use for our islands as they are not single points) to make the initial distance matrix. We tried using our own distance matrices (minimum distance / connectivity matrices), but the analysis ultimately failed to work. We also tried using another analysis strategy called capscale, but that only included one matrix at a time and thus could not compare associations between island characteristics, species similarity, and float times simultaneously.

Regarding the main text, the first thing I find quite confusing is the term "fruited plants", as it broadly means plants with fruits. After reading the manuscript I believe the authors are referring to plants that produce fruits with endozoochorous characteristics in the sense Heleno & Vargas (2015), i.e. edible fruits with fleshy pulp and/or bright colours that attract animals that may eat them. This is the first thing the authors should make clear, as this study is clearly focused on species with characteristics that make them good candidates to be mainly dispersed by animals. Then, I would recommend that the introduction focus first on the dispersal of plants with endozoochorous fruits (hereafter fleshy fruited plants for simplicity), and the problems of defaunation specially in the long-distance dispersal (LDD) of this species. Then I would focus on islands and LDD, and the decoupling between the dispersal syndrome and the dispersal vector (see Heleno & Vargas, 2015). Finally, I would introduced the potential of fleshy fruits to be dispersed by ocean currents (Esteves et al., 2015) that would ultimately lead to the main knowledge gap that this paper addresses and to the main questions. If I have misunderstood the main species selection criterion for this study (i.e. plants producing endozoochorous fruits), the authors should clearly state their criteria for plant selection. As this determines the whole text and its main focus.

See previous comment. We have mainly followed your suggestions for rewriting our Introduction. We start now with a discussion of LDD as you suggest.

I find the methods section a bit confusing because of all the terminology, the complexity of the methods and the many tests used. In particular, from line 235 to line 266, I strongly recommend that the authors make an effort to rewrite it for the sake of clarity. In this paragraph the same or similar terms are used for different things. First, the authors talk about thalassochoric connectivity (L. 235), which is species specific. Then, it is tested the correlation between geographic distance and thalassochoric dispersal potential (L. 242), but this latest concept refers to the two thalassochoric dispersal metrics (Tables S1 & S2), which have nothing to do with the species but with the ocean currents themselves. It continues with the evaluation of the predictive power of the thalassochoric dispersal potential on species distributions (three tests; L. 249), but the two first tests are species specific and they use either the viability metrics or the species connectivity matrices (not the dispersal metrics Tables S1 & S2), and the third test refers to the ocean current connectivity matrices as the dispersal probability and minimum dispersal time (dispersal metrics Tables S1 & S2), not the species thalassochoric connectivity.

We have rewritten parts of the method. Hopefully it is more clear now.

My recommendations for improving the analyses are:
(1) GLM fitted using a Poisson family with the number of islands that each species occur on (species range size) as response variable, and the species viability metrics (maximum / 95th percentile) and human use as predictor variables [no.islands ~ viability + human use]. Instead of the linear regression of L. 250-251 and L. 263-265.

This suggestion was very helpful as it now showed significant relationships. See lines 321-326: “In assessing the capacity of ocean-current dispersal potential for predicting species distributions, we found a significant, positive relationship between maximum and 95th percentile viability time and species range size for all study species (Fig 3: max: z = 2.18, p = 0.03; 95th percentile: z = 8.08, p < 0.001). This was accompanied by a significant, positive relationship between species range size and the sum of human use categories as well (Fig 3: max: z = 2.72, p = 0.006; 95th percentile: z = 3.36, p < 0.001).”

(2) Generalized dissimilarity models (GDM) to investigate the role of geography, ocean currents and island attributes (e.g. human population, island size, island age, climatic values per island) in influencing floristic similarity between islands (Sørensen index) (Ferrier et al., 2007; Mokany et al., 2022). These models allow the use of a floristic similarity matrix as a response variable and a combination of distance matrices and raw data as predictors. In this way, the authors could also assess the influence of island characteristics, such as island size or age, which according island biogeography theory are of great importance in shaping island biodiversity, but also human-related island characteristics such as population density or some island metric of human impact (L. 400-402). At this point, one thing the authors need to be careful about is the high correlation between thalassochoric dispersal metrics and geographic distance. This may affect the GDM results and perhaps it would be worthwhile to additionally fit GDMs with geography (+ island characteristics) and dispersal metric (+ island characteristics) separately.

We tried GDMs, but the package struggles when not using coordinates, and the islands are not single points that can be represented with coordinates. We tried using capscale instead, which is similar but only includes one matrix at a time, so I can see how island characteristics are associated with species similarity but cannot simultaneously include float times.

Regarding the data, particularly Supp. Tables 1, 2, 5 and 6, how have NAs been handled in the analyses? I understand that the connectivity measures with NA values are those with no buoys connecting two islands, which makes me wonder if they have been converted to zero for the probability of dispersal and to a very high number for the minimum dispersal time. In this way, these "unconnected" island pairs are included in the analyses. However, the procedure followed by the authors is not clear in the text, apart from the mention of removing seven islands from the analysis (L. 292-293). Moreover, I do not understand why there are NAs in Supp. Tables 5 and 6 (Sørensen index). If two islands have no species in common, should not it be zero? These concerns should be addressed as they directly affect the results and statistical power of the analyses.

Thank you for catching this. Our initial analysis did treat them as NAs. We have now edited the matrices following the guidance you provided (zero for the probability of dispersal and 999 for the minimum dispersal times). As it turned out, the results were similar to what we’d found previously.

Other comments:

Title: I would recommend the authors to think of a more catchy title, as the one they have proposed suggests that no conclusions are drawn about the role of ocean currents as a dispersal vector, which this is not very appealing.

We changed the title to: “Testing the potential of Caribbean fleshy-fruited plants to disperse using ocean currents”.

L. 35: Replace 2.6 x 10³ km by 2600 km, as it is not a super big number and it will improve readability.

Done

L. 56-57: Ocean currents is a vector, thalassochory is the dispersal mechanism, please rephrase. In addition, there are not many vectors capable to transport propagules to islands, they are either wind, ocean currents or flying animals, so the authors can confidently reformulate the sentence into a statement: instead of "ocean currents are thought", "ocean currents are one of the main LDD vectors".

Done

L. 65: I do not understand what this has to do with the role of ocean currents in connecting populations, I would remove the last part of the sentence "actual connectivity is limited by turbulent dispersal" unless the authors find a better way to integrate it into the flow of the text.

Done

L. 70: I would not say that the ability for fruits to float and disperse is widely documented. I would rather say that it has been documented for some species with special emphasis on coastal species.

Changed from
“this ability for fruits to float and disperse is widely documented, particularly in coastal regions and islands”

to
“Successful thalassochory for fleshy-fruited plants requires the fruit or seed to float and the seed to stay viable while at sea – both of which have been documented for plants in coastal regions and islands” (Lines 90-92)

L. 71-73: Is this referring to long-distance dispersal to islands? If so, there are only three other ways for plants to overpass the sea barrier. Apart from what the authors specify here, propagules can arrive on islands attached to the fur/feathers/skin of flying vertebrates (epizoochory) (e.g. Aoyama et al., 2012). The authors should clarify here if they are talking about LDD to islands and if so, specify all the other ways of LDD for plants to reach islands (anemochory, endo- and epizoochory). And then remove "thalassochory is usually not the only method of dispersal by plants" as it is obvious. I recommend to rephrase the whole paragraph.

The paragraph has been restructured to cover all of the different means of plants to arrive on islands and to better represent the amount of evidence for thalassochory.

L. 116: "main" instead of "mean"?

Changed

L. 127: why relying on existing dataset if the authors worked directly with the fruits? Why not measuring them before the experiments?

We agree that this would have been a better methodology, but instead traits were measured from other fruits that came from the same living collections.

Table 1: it would be interesting to include the maximum number of days the fruits were floating per species. I am wondering if this value is decoupled with seed viability in some species, for example, due to aborted seeds, these "empty" seeds will favour higher fruit floatability.

In all but one case, the maximum number of days that seeds remained viable and the maximum number of days that they remained floating were identical. We have added the following text to the Results to convey this information: “Most of the species remained viable until they sank. The only exception was *T. jussieui*, in which the last individual sank after 28 days and the last time seeds were shown to be viable was after 21 days.” (Line 304-306).

L. 190: "[" 33, 46, 47].

Changed

Figure 2 should indicate the species to which each curve belongs. In my opinion, the colour would not be necessary if an horizontal line indicating the 95% of floating viable fruits is included. The y-axis should be labelled "Percentage of viable floating fruits". If the authors have chosen to present the x-axis in logarithmic scale only for better visualization of all lines, this should be mentioned in the figure caption. I have a suggestion regarding this x-axis transformation, it is true that it would be difficult to differentiate all the early sinking species lines in a natural scale, but it is also easier to understand. So my suggestion is to do a first plot with the x-axis in natural scale and then do either a zoom in and show only a subset of days (e.g. 10 or 20 days) or show a second plot with the x-axis in logarithmic scale.

We have edited Figure 2 following your suggestions. It now includes a legend that can be used to identify the curves for each species and a horizontal line illustrating when only 5% of fruits are left floating. We decided to keep the x-axis on a log-scale, but to express the values along this axis in their original units in order to ease interpretation.

L. 250: "we conducted a linear regression", if the response variable was the number of islands (which should be indicated), this variable follows a Poisson distribution, and hence a GLM should be performed instead.

We have updated the analysis as you suggested.

L. 311: "(insert stats)"

We apologize for this oversight and have now included the correct statistics based on the updated method (generalized linear mixed model with Poisson error distribution). See lines 324-326: “This was accompanied by a significant, positive relationship between species range size and the sum of human use categories as well (Fig 3: max: z = 2.72, p = 0.006; 95th percentile: z = 3.36, p < 0.001). “

L. 318-319: already mentioned in L. 309-311.

Removed

Figure 3: x-axis label should indicate that it represents the maximum viability time. Why the square root? If this value was transform for fitting the model, it should be specified in the main text. Y-axis label should be "Log (number of islands)", and specify in the figure caption that number of islands is the "range size". Was the logarithmic transformation only for representation purposes only?

We have updated Figure 3 so that the y-axis is spaced on a log scale, but the values are expressed in their original units for ease of interpretation. We similarly space the x-axis on a square root scale, but again express values in their original units. We now specify in the main text that this transformation was used when fitting the model. We have also updated both axis labels to ease interpretation of the figure.

L. 332: I would avoid the term 'adapted' as it implies an evolutionary relationship between a particular biotic/abiotic factor and a species trait. In this case, we do not know whether the higher floatability and viability of certain species has evolved in response to a more efficient sea dispersal, or whether it is an unintended consequence of the particular evolutionary history of the species. I would recommend using "better suited for dispersal by ocean currents" instead.

Changed

L. 378: "hollow seeds" can be considered as seed with an air chamber, in line with Heleno & Vargas (2015) (L. 369-371), then it would contradict the statement made in L. 371-372.

Rearranged to paragraph to state that *C. icaco* seeds are associated with the characteristics suggested by Heleno and Vargas.

Supp. Table 7: I could not find it in Github (<https://github.com/sxk1332/floating_fruits/tree/main/Supp_materials>)

Added

References
Aoyama, Y., Kawakami, K., & Chiba, S. (2012). Seabirds as adhesive seed dispersers of alien and native plants in the oceanic Ogasawara Islands, Japan. Biodiversity and Conservation, 21(11), 2787–2801. <https://doi.org/10.1007/s10531-012-0336-9>
Calsbeek, R., & Smith, T. B. (2003). Ocean currents mediate evolution in island lizards. Nature, 426(6966), 552–555. <https://doi.org/10.1038/nature02143>
Esteves, C. F., Costa, J. M., Vargas, P., Freitas, H., & Heleno, R. H. (2015). On the Limited Potential of Azorean Fleshy Fruits for Oceanic Dispersal. PLOS ONE, 10(10), e0138882. <https://doi.org/10.1371/journal.pone.0138882>
Ferrier, S., Manion, G., Elith, J., & Richardson, K. (2007). Using generalized dissimilarity modelling to analyse and predict patterns of beta diversity in regional biodiversity assessment. Diversity and Distributions, 13(3), 252–264. <https://doi.org/10.1111/j.1472-4642.2007.00341.x>
Heleno, R., & Vargas, P. (2015). How do islands become green? Global Ecology and Biogeography, 24(5), 518–526. <https://doi.org/10.1111/geb.12273>
Higgins, S. I., Nathan, R., & Cain, M. L. (2003). Are long-distance dispersal events in plants usually caused by nonstandard means of dispersal? Ecology, 84(8), 1945–1956. <https://doi.org/10.1890/01-0616>
Hodel, R. G. J., Knowles, L. L., McDaniel, S. F., Payton, A. C., Dunaway, J. F., Soltis, P. S., & Soltis, D. E. (2018). Terrestrial species adapted to sea dispersal: Differences in propagule dispersal of two Caribbean mangroves. Molecular Ecology, 27(22), 4612–4626. <https://doi.org/10.1111/mec.14894>
Miryeganeh, M., Takayama, K., Tateishi, Y., & Kajita, T. (2014). Long-Distance Dispersal by Sea-Drifted Seeds Has Maintained the Global Distribution of Ipomoea pes-caprae subsp. Brasiliensis (Convolvulaceae). PLOS ONE, 9(4), e91836. <https://doi.org/10.1371/journal.pone.0091836>
Mokany, K., Ware, C., Woolley, S. N. C., Ferrier, S., & Fitzpatrick, M. C. (2022). A working guide to harnessing generalized dissimilarity modelling for biodiversity analysis and conservation assessment. Global Ecology and Biogeography, 31(4), 802–821. <https://doi.org/10.1111/geb.13459>
Nathan, R. (2006). Long-Distance Dispersal of Plants. Science, 313(5788), 786–788. <https://doi.org/10.1126/science.1124975>

Reviewer #3: This study examines how long (if!) the fruits of some fleshy-fruited species in the Caribbean stay afloat in saltwater, and what this means for seed viability. This is an important question for long-term vegetation dynamics in any island-rich region – and thus especially so in the Caribbean, with its myriad islands and archipelagoes.
I really liked that the authors used two complementary methods to investigate floating potential (even if not possible for all species); this is done much too rarely in such studies.
While topically interesting and with valuable data, the study in its current form suffers from several major issues:

1) The most important issue is the phylogenetic non-independence in your data: A whopping ten out of the 14 study species are from the same family (Arecaceae; palms), and three of these from the same genus. Given how similar the fruits of many palm species are, this strong non-independence of your study species needs to be discussed (in terms of how it could/could not impact your results & their interpretation).
This is especially important for your analyses, where you should really include family (or at least genus) as a random effect (or at least fully explain why this is not necessary).

Thank you for this suggestion. We have now included family as a random effect in our GLMs.

2) I don’t buy the argument that thalassochory is becoming increasingly important in light of the terrestrial defaunation of large-bodied dispersers. The former is most concerned with inter-island dispersal, while the latter is almost entirely about intra-island dispersal.
For the day-to-day ecology of any slightly large island (where ‘inland’ > ‘coastal’ ecosystems), what matters most is the loss of (formerly common) intra-island dispersal, rather than the maintenance of (comparatively uncommon) oceanic transport of seeds from fleshy-fruited plants. I would suggest toning down the quite strongly worded conservation implications for island ecosystems in general, and stick to what it means for coastal plant communities.

We toned down conservation aspects of our narrative and instead focus more on how we are examining thalassochory as an alternative means of dispersal for fleshy-fruited plants that primarily use endozoochory. We also framed our introduction more with how our work fits in with understanding long distance dispersal (LDD).

3) There are some issues with the origin and use of ‘traits’ in the MS that need to be addressed. The authors give [35] as reference for the size and weight (“We relied on an existing dataset [35] for information regarding the size and mass of each fruit.”) – but this reference is an in-prep study by an author not on the current MS, and should not be used/cited in this way.
Please cite these measurements as unpublished data, and make sure to acknowledge this person for the use of it. (and see my point 5, below, for how this affects Table 1, too).

We have included the primary author of the dataset on fruit size and weight as a coauthor and have provided a brief summary of how these data were collected since the dataset is not yet published. See line 185-190: “This involved consulting an existing database of fruit traits [40] which used digital calipers and scale to measure relevant traits of at least 10 specimens from 259 fruiting plant species native to the Caribbean archipelagos that were obtained from Montgomery Botanical Center, the Gifford Arboretum at the University of Miami, Fairchild Tropical Botanical Garden, Biscayne National Park, and various field collections in several Caribbean islands (Virgin islands, Lesser Antilles, Bahamas, etc).”

4) Throughout the MS the writing is quite ‘loose’/unclear when it comes to the use of ‘plant’, ‘species’, and ‘fruit’. I have tried to catch the worst of them & suggest rewordings (in the section on minor issues, below), but please make sure that you are as specific as possible, to make it easier for the reader to follow your arguments. In particular, use ‘fleshy-fruited’ instead of ‘fruiting’.

We have removed any references to “fruiting” and changed them to “fleshy-fruited”.

5) Table 1 has several issues that need to be addressed:
- Please add a column with family of the species; to many readers this will be much more informative to get a quick idea of the fruit type (e.g., that ten of the 14 study species are palms!) than only giving the speices name.
- It is unconventional to rank study species in the methods by results given in the results section; It should really be done by plant family (& alphabetically within family) as is usual for such studies.
- For anyone looking at Table 1, they will currently almost certainly make the assumption that these measurements come from the fruits you used in your study (column 5, ‘initial count’). Please make sure to include the info for the origin on these trait values in the legend, too.
- It makes no ecological sense to give the mean values in columns 2-3 with an accuracy of two decimal points, please simplify to one.
- The title is a bit misleading, since only 3 out of 7 columns are actualy traits; I suggest renaming this to simply say ‘Study species’.

We have added the column of families and reorganized the table based on taxonomy. We have also provided information on the source of the trait data, corrected the number of decimal points, and edited the title.

6) Figure 2 has some issues that need to be addressed:
- The legend: While you use survival curves to show your results, ‘survival’ is not what you measure/show here, but rather floating capability. Also, the first line reads ‘of all species used in our study’, but the last line says that the seeds of four that sank within a day are not given.
Please change the first line to something more meaningful/simple, e.g., “Fig 2. Floating capacity of fruits”.
- Giving each curve a colour based on the floating capacity is odd, as this information can be read/seen directly in the figure, anyway. This also currently hides the biological meaningful information about which survival curve belongs to which species.
Instead, please give each species’ curve a different colour and label the individual survival curves – e.g., with a number somewhere along its length – and list the corresponding species in the legend (or to the right of/below the main figure).

We now provide a legend so that the curve for each species can be identified. We have renamed the figure as you suggested.

7) Throughout the MS, I suggest simplifying ‘fruit traits’ to simply be referred to as ‘size and weight’ (as these are the only ones you recorded; for many dispersal ecologists, ‘fruit traits’ usually refers to more than just these two.

We have made these changes.

8) L311 – ‘(insert stats’) – yes, please!

Removed altogether as we did a new analysis at the suggestion of another reviewer. See lines 324-326: “This was accompanied by a significant, positive relationship between species range size and the sum of human use categories as well (Fig 3: max: z = 2.72, p = 0.006; 95th percentile: z = 3.36, p < 0.001). “

Minor issues and corrections:
L27 & again in L55 – suggest using a simpler wording, “Thalassochory, or dispersal by ocean currents, …”

Changed

L29-32 – a very long and winding sentence, suggest splitting up into two.

Done

L30 – correct ‘fruiting’ to ‘fleshy-fruited’.

Done

L31 – insert comma, “…potential, and…”

Split into two sentences instead.

L33 – you have just defined which plants you study in the previous sentence; hence suggest simplifying here by deleting, “…of plants in the region”.

Done

L34 – suggest using, “…feasibly being able to float…”

Done

L35 – there is no need to use exponential notation here, just say 2,600 km…

Done

L36 – reword to, “...for fruits with longer floating times…”

Done

L43 – correct to ‘archipelagos’, to fit with usage in rest of MS.

Done

L44 – insert comma, “…saltwater, and 2)…”

Done

L45 – correct ‘fruiting’ to ‘fleshy-fruited’.

Done

L52 – reword to, “…and have implications for both conservation management and biogeography”.

Done

L52 – correct to, “Islands are classic study systems…”

Removed as part of a restructuring of the paragraph.

L69 – This sentence is very unclear/uses wrong terms – suggest rewriting to, “Succesful thalassochory requires the fruit or seed to float and the seed to stay viable while at sea – both of which have been widely documented for plants in coastal regions and islands”, or similar.

Removed as part of a restructuring of the paragraph.

L75 – there is only species in the genus Cocos; correct to ‘Cocos nucifera’)

Done

L76 – insert comma between ‘dispersal and’.

Done

L79 – correct to ‘large-fruited species’

Done

L83 – correct ‘fruiting’ to ‘fleshy-fruited’.

Done

L84 – insert comma after ‘dispersal [27]’

Done

L90 – insert comma after ‘…than others’

Done

L93 – insert comma after ‘archipelagos’

Done

L94 – simplify to, “We address the following…”

Done

L94 – reword to, “Do the fruits of some plant species…”

Done

L96 – correct ‘fruiting’ to ‘fleshy-fruited’.

Done

L98 – reword to, “…plants with fruits that float better…”

Done

L110 – correct to ‘eddy-dominated’

Done

L112 – insert comma after ‘gyre’.

Done

L129/280/282 – please use ‘records/recorded’ instead of ‘observations/observed’ (also rename corresponding column in Table 1 to reflect this)

Done

L173 – correct ‘fruiting’ to ‘fleshy-fruited’.

Done

L250 – move contents in parenthesis to appear after ‘…viability metrics’.

Done

L251 – delete ‘size’; species range is already a spatial measure on its own.

We kept this one as we had it, because we did specifically mean the size of the species range and not just its location.

L257 – insert comma after ‘…days)’

Done

L262 – insert comma after ‘genus level’

Removed as a restructuring of the paragraph

L263 – correct to “each species’ range size”

Removed as a restructuring of the paragraph

L263 – delete the first ‘by’ and insert commas before and after ‘first’, to read, “We did so, first, by…”

Removed as a restructuring of the paragraph

L264 – insert commas before and after ‘second’.

Removed as a restructuring of the paragraph

L276 – correct ‘fruiting’ to ‘fleshy-fruited’.

Done

L304 – correct to ‘more strongly correlated’

Changed segment to be more succinct.

L327 – substitute ‘via’ for ‘through’.

Done

L329/L333/L374/L384 – correct ‘fruiting’ to ‘fleshy-fruited’.

Done
