# Supplementary material for: Testing the oceanic dispersal potential of Caribbean fleshy-fruited plants
Source: PLoS One. 2026 May 27;21(5):e0348628. doi: 10.1371/journal.pone.0348628 (PMC13215481; doi:10.1371/journal.pone.0348628)
Supplement: Attachment — Submitted filename: Response to Reviewers3.docx [file pone.0348628.s015.docx]

Seokmin Kim

Reviewer Responses

PLOS One

January 31, 2025

Reviewer #1: I feel the authors satisfactorily addressed the concerns raised by the reviews which contributed to a clearer and stronger manuscript. I therefore believe the manuscript is valid contribution to the field and I would like to congratulate the authors for their nice study. I have only a few specific points for consideration that I outline below.

Thank you for your comments and support. We appreciate you helping improve this manuscript ad hope that our new responses are satisfactory.

Specific comments
L85-86, I’m not sure if the 3 references cited in this sentence actually “demonstrate” that this mechanisms work for inter-island seed dispersal as neither of them managed to record the origin, vector and arrival of o colonizing propagule. We know that these 3 mechanisms are the potential ones because there are the only ones that can promote these movements, because they have been demonstrated to work on another contexts (not between islands), and because there is indirect probabilistic evidence suggesting that they are likely effective vectors. Please revise the term “demonstrated”, and also check that the references are the correct one (e.g. the paper dealing with the dispersal of the coconut palm is indicated as a demonstration of epizoochory). Also correct “..”

Changed “demonstrated” to “There are other mechanisms by which plants can disperse between islands such as anemochory – wind dispersal, [27] and epizoochory – attachment to feathers, fur, or skin [28].” (Line 86-87) Changed reference #28. Removed the extra period.

L96 The concept of “artificial introductions” is unclear. Consider replacing with anthropogenic introductions or a similar term.

Changed to “anthropogenic introductions”

L284 correct “..”

Corrected

L309 consider: visit -> visiting

We kept the original wording for this.

L435-443 I’m not fully convinced that “growth rate” is a particularly informative variable to include in future studies. Even if plants exposed to salty environments grow more slowly due to physiologic constraints, they can still promote gene flow and new colonization events (only slower). Moreover, as the current manuscript is not about growth rates, this seems like a bad choice of topic to close the manuscript.

The goal of our study is to determine whether thalassochory could replace endozoochory for plants that lack extant animals large enough to effectively disperse them. Thus, while it is true that as long as any viable seed can make it from one island to another, that thalassochory can contribute to the dispersal of that species, the degree to which thalassochory can replace endozoochory is affected by all other influences of saltwater exposure on seed fitness. Thus, we feel that growth rate after germination is a relevant demographic metric to track in future work.

Reviewer #2: The revised version of the manuscript, now entitled "Testing the potential of Caribbean fleshy-fruited plants to disperse using ocean currents" by Kim et al., has improved compared to the first version I read. I appreciate the effort made by the authors in addressing all of the reviewer's concerns. However, I believe further work is still needed on building the background to provide a clearer framework for the study, and on presenting the statistical results.

More care should be taken in relating the topics of the effect of defaunation on fleshy fruit dispersal and the long-distance dispersal (LDD) of plants. Long-distance dispersal is a rare process in nature compared to dispersal over shorter distances (long tail of the species' dispersal kernel). Different dispersal vectors may play a role in this process, and these vectors do not necessarily align with plant dispersal specializations (non-standard means of dispersal, see e.g. Nathan 2006; Nogales et al. 2012; Vargas et al. 2012). This decoupling between the plant dispersal syndrome (in this case, fleshy fruits, that are indicative of an endozoochorous syndrome) and the dispersal vector opens the door to investigating how well suited are plant diaspores to be dispersed by non-standard vectors (e.g. fleshy fruits dispersed by sea). However, throughout the text, the authors sometimes give the impression that they are referring to dispersal as a deterministic process (e.g. L. 341-342), and that plants only look for another appropriate vector when the standard vector is missing (e.g. sea dispersal as an alternative to endozoochory in the absence of frugivores). I find the defaunation phenomenon an interesting motivation to assess the ability of fleshy fruits to be dispersed by other means, like sea dispersal. However, stronger evidence of the effect of the decline of frugivores on LDD, especially on islands, is needed. As another reviewer pointed out, defaunation may pose a significant challenge to inland plant dispersal (over short-medium distances) but not to crossing the sea barrier between islands (LDD). In line with my suggestion about LDD and plant dispersal syndromes, the authors can use the introduction of Esteves et al (2015), where the authors assessed the potential of Azorean fleshy fruits to be dispersed by sea currents, as a reference.

Thank you for your detailed suggestions. Regarding the introduction, we have rearranged the beginning of it so that it now starts by discussing dispersal, connectivity, and LDD. We then explain why endozoochorous, fleshy-fruited plants are interesting within this context and elaborate on how these have been affected by defaunation. Next, we explore how fleshy-fruited plants could also utilize thalassochory as a LDD mechanism before going onto the rest of the introduction as before.

Another concern I have, especially after reading the Discussion, is the use and interpretation of 'population connectivity' in the framework of this study. This study is looking at the species presence/absence in the Caribbean islands, which must indeed be the result of some connection between islands. However, this does not mean that the connection is permanent or detectable nowadays. For example, an island could be colonized by a plant species after a single successful event of LDD followed by a complete isolation. Alternatively, a different regime of sea currents in the past might have connected two currently isolated islands. Presence/absence data cannot rule out the role of sea dispersal (or any other kind of dispersal) in island colonization, even if no correlation is found between species distribution and sea connectivity. Please, discuss with caution the topic of population connectivity, particularly when comparing the results of this study with the results of population genetic studies where gene flow gives a measure of the intensity of inter-island population connectivity. **It should be clear in the Discussion that the species distribution data does not reflect connectivity per se, but rather at least one successful event of island colonization. This can be argued as the reason behind the absence of significant correlation between species distribution and sea connectivity.**

We added the following in lines 429-433 “. It only takes a single successful LDD event to colonize a new island [5, 6]. The rare and stochastic nature of such events may make it difficult to correlate their outcome (species distributions across islands) with mean connectivity metrics, whether they be purely distance based or measured empirically based on ocean currents.” We hope that this clarifies our discussion with regards to your concerns.

The presentation of the statistical analysis' results in the manuscript should be improved, particularly of the (generalized) linear mixed models. The authors fitted LMM and GLMM sometimes using more than one predictor variable. However, they do not mention how well the models fit the data. What did the residuals of the models look like? Were there any violations of the model assumptions? Did any of the models experience convergence or singularity issues? Did adding a random factor improve the models? Given the small amount of data (only 14 species), I am aware that the models will not be perfect, and I suspect that the most complex ones might have problems. This should be shown and discussed in the manuscript. Thus, I recommend that the authors: (1) check the residuals of the models and mention this in the text; (2) compare each fitted models with simpler ones (first testing the random structure and then the fixed effects), and interpret the best model; (3) show the estimate for each predictor variable, not just the result of the statistical test, as the estimate specifies the strength and sign of the effect on the response. All of this will make the analyses more robust and reliable. There are quite a few papers and books explaining GLMMs and best practices; here, I include just two references the authors might find useful: Harrison et al (2008); Bolker et al. (2009).

In our revision, we checked residuals and convergence/singularity and found no issues. We also added estimates and their standard error into the text and state how there were no serious issues with model assumptions. See line 338-344: “(Fig 3: max: estimate (standard error) = 0.19 (0.086), z = 2.18, p = 0.03; 95th percentile: estimate (standard error) = 0.25 (0.031), z = 8.08, p < 0.001). This was accompanied by a significant, positive relationship between species range size and the sum of human use categories as well (Fig 3: estimate (standard error) = 0.22 (0.082), max: z = 2.72, p = 0.006; 95th percentile: estimate (standard error) = 0.25 (0.075), z = 3.36, p < 0.001). Residual plots against predicted values for both sets of models showed no systemic patterns, and none of the models experienced convergence or singularity issues.”

Regarding comparisons between fitted models and simpler ones, models with and without random effects were within a delta AIC of 2. Removing the random effect did not change the significance of any of the fixed effects. While the model without the random effect had a lower AIC, due to our concern about accounting for phylogenetic non-independence, we reported the model with the random effect even though the AIC was marginally higher.

Here, I detailed some suggestions focused on the different sections of the manuscript:

Abstract: It gives the reader a clear idea of the main question, methods and results. However, rephrasing the final part would make it more engaging. I would recommend splitting the long sentence that mixes results and conclusion about the drivers of plant distribution (L. 39-43), and separating the results (no relationship between geographic distance and species distribution; human usage) from the main conclusion ("challenge associated with identifying drivers of distribution ..."). I would emphasize this final part, as it is the main message derived from the association analysis. I would also shorten the final sentence on future studies to convey that, although there are still open questions, this study is the necessary first step in understanding the role of sea currents in the distribution of fleshy-fruited species.

We changed lines 39-46 to “Geographic distance, the isolation metric traditionally used in island biogeography studies, could not explain distribution or community patterns either, while human usage was identified as an alternative significant predictor of species range size. Our results thus illustrate the challenges associated with identifying drivers of distribution patterns across the Caribbean archipelagos. While we assert that future studies, such as those examining genetic connectivity of plant populations in the context of thalassochoric connectivity, are needed, this study serves as a crucial first step in understanding the role of sea currents in the distribution of fleshy-fruited plants.”

Introduction: The structure of the Introduction can be better improved, in the current version, I get the impression that different topics are introduced without being clearly connected with each other (defaunation, islands, LDD).

We have rearranged the beginning of it so that it now starts by discussing dispersal, connectivity, and LDD. We then explain why endozoochorous, fleshy-fruited plants are interesting within this context and elaborate on how these have been affected by defaunation. Next, we explore how fleshy-fruited plants could also utilize thalassochory as a LDD mechanism before going onto the rest of the introduction as before.

Methods and results: I appreciate the effort the authors have made to improve the clarity of this part of the manuscript. However, I still find a bit confusing the last part of the Methods section. From line 253 to the end of the section, the authors describe different analyses one after the other in a way that might be confusing for the reader. I suggest introducing clearly the different analyses using a brief description of the motivation behind. Dividing the section into two, for example "dispersal potential and species connectivity calculation" and "statistical analysis" might benefit the reading of this section.

We separated the methods into two sections as suggested.

Regarding the authors' attempt to use GDMs, as commented in their response to reviewers, it is indeed necessary to include coordinates in the input as instructed in the manual. However, if the 'geo' option in the gdm() function is set to 'FALSE', geographic distances are not calculated from the provided coordinates. The authors should therefore run the function with fake coordinate columns and provide the geographic distance matrix together with other predictor variables (e.g. island characteristics; be careful if including sea connectivity in the same model as geographic distance because their high correlation), and set 'geo=FALSE'. This way it should run smoothly. Maybe it is not that interesting fitting GDM for each species independently, as there should be many NAs in most of them. However, it might be worthwhile implementing them with Sørensen matrices as the response (non-floating and floating community dissimilarity).

Thank you for your suggestion. I did not consider providing fake coordinate columns. That allowed me to use the “formatsitepair” function, which I was previously unable to use. However, we are still getting errors in the final step despite putting geo = FALSE.

*Error in gdm(sitePairTable, geo = FALSE) :*

*NA/NaN/Inf in foreign function call (arg 8)*

*In addition: Warning messages:*

*1: In mean.default(sort(x, partial = half + 0L:1L)[half + 0L:1L]) :*

*argument is not numeric or logical: returning NA*

*2: In gdm(sitePairTable, geo = FALSE) : NAs introduced by coercion*

We get this despite making sure there aren’t NA values in our dataframes.

With some other dataframes, we also get this error. We then checked to make sure the matrices had the same number of rows and columns, but we still got the same error.

*Error in createsitepair(dist = distData, spdata = bioData, envInfo = predData, :*

*The length of distance values is not the same as the expected number of rows in the site-pair table, unable to proceed.*

Given the difficulties of running this model, we decided not to include GDMs in our manuscript.

Minor comments:
L. 57 & 58: use the same type of dash to indicate the break in the sentence structure.

Changed.

L. 64: the Rey & Alcántara (2000) ([11]) reference is about a single fleshy-fruited species (Olea europaea), and it is not sufficient on its own to support the authors' general statement about the importance of frugivorous seed LDD on isolated ecosystems. Please find additional papers that support this general statement.

Merged citations so that they are listed at the end of the sentence. Additionally, added Carlquist (1967) and Vargas et al. (2014) as references.

L. 85 & 86: semicolons before the references should be removed. Also, replace the hyphens by either "or" or parentheses.

Fixed

L. 85: the Costea et al. (2019) ([25]) study does indeed focus on endozoochory, but not driven by frugivorous birds. Instead, they demonstrate the important role of granivorous birds on plant dispersal. Please either find a more suitable reference or rephrase.

We changed this sentence in response to a recommendation from Reviewer 1. Endozoochory is no longer mentioned here.

L. 86: the Harries & Clement (2014) ([27]) reference is about the coconut palm and it does not reference epizoochory.

Reference changed

L. 86: the extra period at the end of the sentence should be removed.

Removed

L. 90-92: from this sentence, I understand that the authors are talking about thalassochory in fleshy-fruited plants. However, the references [28-30] are either general references assessing different species [28 & 30] or focused on a single non-fleshy-fruited species [29]. Please rephrase to improve the clarity of the reasoning here.

Removed “fleshy” and added “propagule”. See line 91-94: “Successful thalassochory for fruited plants requires the propagule (fruit or seed) to float and the seed to stay viable while at sea – both of which have been documented for plants in coastal regions and islands [30-31].“

L. 105: remove the comma before "(2)".

Removed

L. 114-125: Please include the number of islands in the study area. Knowing the total number of islands would give the reader a better idea of how well each species is distributed when looking at Table 1 data.

We mention how our study area has 44 major islands first in line 219-220. (“Our study area was between 10.3 and 27.4 latitude and -86.3 and -58.8 longitude, which encompasses all 44 islands within our study”) However, we agree that it would be better to include this information earlier by inserting this sentence at line 117-118: “For our study, we considered 44 major (here considered as those larger than 100 km2) islands within this region.“ Additionally, we edited the former sentence to state: “which encompasses all 44 islands within our study.”

Table 1: Is 'initial count' the total number of fruits? If so, please specify. Also, be careful with the formatting. For example, the Chrysobalanus icaco row has a different font size and alignment.

We changed the table column name to “total count” and revised the legend to include “total fruit count used in the study”

Figure 2: What does it represent, the floatability or the viability of the seeds? Based on Table 1 data, I do not understand why Chrysobalanus icaco does not reach the 95th percentile line in the plot, and why some species do not reach zero. Please clarify it in the Figure caption.

The previous figure just represented the floatability. Reconsidering it, we decided to change the figure to a new one that represents viability instead. Since this incorporates both floatability and the fruits’ ability to survive, we think this better summarizes the full results of our study.

L. 182-183: I guess the authors did some extrapolation combining data of the percentage of floating fruits obtained twice a week and the percentage of viable seeds obtained once a week for obtaining the curves showed in Fig. 2. More detail is needed to be able to follow how the authors did this calculation. Does the y-axis in Fig. 2 represent the percentage of floating fruits instead of viable floating fruits?

That is correct. We assumed that the proportion of seeds still viable in our floating apparatus were the same as the proportion of seeds shown to be viable through the Tetrazolium test. For example, if we found that half of the samples that underwent the Tetrazolium test were viable, we considered that half of our sample were viable as well. We added this sentence in lines 182-183 to clarify this: “We assumed that the proportion of viable seeds in our floating apparatuses were the same as the proportion of seeds shown to be viable through the tetrazolium test.”

L. 190-192: Which is the response variable? Because I understand the question as the influence of fruit characteristics on the ability to float and survive in sea water. Thus, I would say that the response should be the maximum or the 95th percentile viability, and the predictors the fruit volume and density (check for collinearity). However, I understand the opposite from the text. Please clarify.

Changed to “We then conducted linear mixed regressions with one of the two (maximum and 95^th^ percentile) viability metrics as the response variable and fruit volume or density as the independent variable, with plant family as a random effect through the *lme4* R package [48].” Lines 193-196.

L. 254-259: Please clarify the terms of the formula. Is plant survival a percentage of viable floating seeds at a given time based on different 'Buoy time'? This 'Buoy time' is the transit time of each of the shared buoys between two islands? If so, why 'n' is not the number of shared buoys? Is connectivity the proportion of fruits that could successfully move between each island pair?

To clarify this, we included the following in lines 263-265: “Plant survival” is the percentage of viable floating seeds, “Dispersal” is the fraction of buoys from the source island that reaches the destination island, and “${Time}_{n}"$ is the number of days ranging from 1 – 90 days (the time scale of our study).” Please note that we changed from “Buoy time” to “Time.

L. 267: What R package was used for fitting the GLMM?

We used the “lme4” package in R. We have now cited this accordingly.

L. 279: "each species' distribution matrix", please specify the number of species with which this analysis was performed. In Table 1, it is shown that some species are distributed only in 1-2 islands, and I presume that these were removed from the analysis.

We considered any species with greater than 0 maximum viability time (ten species). We figured that those with small distributions would provide non-significant results, but we included them anyway for the sake of being thorough. We have now added the following to clarify this. See line 285-286: “…for the ten species that had greater than zero maximum viability times.”

Figure 3: Another plot can be included showing the effect of human usage in species distribution, as this predictor was also significant in the model.

Plot made and included as part of figure 3. Changed the caption to now read: “Fig 3. Both oceanic dispersal capacity and the degree of human use were significant predictors of species range size (Maximum viability: z = 2.2, p = 0.03, Sum of human usage: z = 2.7, p = 0.006). Range size (number of islands on which a species occurred) is shown on a log-scale, although the model was fit using the integer values and a Poisson error distribution. Maximum viability time was square root-transformed to increase normality.”

L. 328: "or geographic distance", there is no reference to a correlation test between species distribution and geographic distance (L. 274-276). Same for L. 332 (L. 277-282 in Methods).

We have added the following in lines 292-294: “We repeated the second and third steps with geographic distance between island pairs instead of connectivity metrics to assess any correlations between geographic distance and species distributions.”

L. 328-330: If in L. 330-332 the authors explain the results of the correlation test between species similarities of non-floating and floating communities with metrics of ocean dispersal, what is this previous result? In methods is no other correlation test assessing the relationship of other community similarity matrix with dispersal metrics.

In lines 287-292, we state the following: “Third, we created two community similarity matrices using the Sørensen similarity index for species that are either good floaters (S5 Table) or poor floaters (S6 Table) based on whether they can float for more than the median 95th percentile viability time of all study plants (7 days), and assessed correlation between these matrices and the thalassochoric dispersal metrics (dispersal probability matrix and dispersal time matrix) using the Spearman’s Mantel test.”

L. 366: Using geographic distance in biogeographic studies provides a reliable objective measure of isolation, and it makes sense that wind or oceanic currents are correlated with the geographic distance. Therefore, in my opinion, it seems somewhat pretentious to claim that "our results largely justify the usage of geographic distance in biogeographic studies".

We have rephrased this to “our results largely agree with the usage of geographic distance as the standard isolation metric in biogeographic studies.” (line 385-386). We agree that the usage of geographic distance in island biogeography does not need to be justified. We only meant that our results were in agreement with it.

L. 372-374: Do the data show evidence of this? I am thinking of species that, although they are theoretically able to reach one island from another by their capacity to float for the estimated minimum time required for colonization, are absent from the island and have a low probability of dispersing between the two islands. This would support the discussion in this paragraph; otherwise, I do not see the interest of this discussion, as no differences were found in the correlation analyses using Sørensen dissimilarity matrices with either dispersal metric. A single successful LDD event between islands might have been enough for a species to colonize and establish on an island.

We believe that this is still interesting to include as we want to emphasize the potential for there being connectivity barriers between geographically close islands.

L. 387: The fact that C. icaco possesses hollow seeds does not reduce the density of the whole fruit? How did the authors calculated fruit density? Using only the pulp? This should be clearly described in Methods.

We calculated fruit density by dividing the mass of each species’ entire fruit (including both pulp and seed) by its volume. We now clarify this in line 137-138 (“…regarding the size and mass of each species’ fruit (defined as the entire fruiting body).” We then detail the exact steps used in lines 186-188 (“We considered each fruit as an ellipsoid and used fruit width and length to calculate its volume. We then divided mass by volume of the entire fruit to calculate its density.”)

L. 400: Please use with caution the term 'adapted' as it implies the action of selective forces in a certain direction. It should be noted that the ability to float and survive in sea water might not be the intended adaptation, but rather a consequence of trait evolution towards other processes/functionalities (e.g. dispersal by birds, protecting the seed, etc.). It is preferable to use 'better suited' instead.

Changed

L. 408: As commented before, it is not mention in Methods that geographic distance was used to evaluate its correlation with species distribution.

Corrected. See previous response.

L. 409-410: A larger number of species must be used to claim this. With 14 species and most of them distributed in less than 10 islands out of 44, the similarity matrix used must not be representative of the real flora similarities between islands.

We agree that our relatively small sample size is a limitation of this study, and we mention this as a caveat in our manuscript. See lines 422-425 (“It is important to note, however, that ocean current dispersal metrics were not effective in predicting distributions for any of our study species, and while we did find a significant relationship between species range size and oceanic dispersal capacity, we caution against making definitive conclusions given our small sample size.”) and lines 449-451 (“While we again caution against making any generalizations given our relatively small sample size, the relationship between plant range size and human usage presents an interesting topic of future study.”)

References:
Nathan, R. (2006). Long-distance dispersal of plants. Science, 313(5788), 786-788. DOI: 10.1126/science.1124975.
Nogales, M., Heleno, R., Traveset, A., & Vargas, P. (2012). Evidence for overlooked mechanisms of long-distance seed dispersal to and between oceanic islands. New Phytologist, 194(2), 313-317.
Vargas, P., Heleno, R., Traveset, A., & Nogales, M. (2012). Colonization of the Galápagos Islands by plants with no specific syndromes for long‐distance dispersal: a new perspective. Ecography, 35(1), 33-43. <https://doi.org/10.1111/j.1600-0587.2011.06980.x>
Esteves, C. F., Costa, J. M., Vargas, P., Freitas, H., & Heleno, R. H. (2015). On the limited potential of Azorean fleshy fruits for oceanic dispersal. PLoS One, 10(10), e0138882. <https://doi.org/10.1371/journal.pone.0138882>
Harrison, X. A., Donaldson, L., Correa-Cano, M. E., Evans, J., Fisher, D. N., Goodwin, C. E., ... & Inger, R. (2018). A brief introduction to mixed effects modelling and multi-model inference in ecology. PeerJ, 6, e4794.
Bolker, B. M., Brooks, M. E., Clark, C. J., Geange, S. W., Poulsen, J. R., Stevens, M. H. H., & White, J. S. S. (2009). Generalized linear mixed models: a practical guide for ecology and evolution. Trends in ecology & evolution, 24(3), 127-135.

Reviewer #3: I want to commend the authors on a very thorough revision that has greatly improved the study. I only have a few suggestions for a few further improvements (Note: All line numbers are for the version ‘Revised Manuscript with Track Changes’!):
Title: The new title is much better, but could potentially be improved even further (the current one puts major emphasis on the ocean current part of the study, which I find a bit of a shame, given the wonderful experimental flotation part of the study). Perhaps something like, “Testing the oceanic [or thalassochoric] dispersal potential of Carribean fleshy-fruited plants” – which would encompass both aspects into one (flotation + currents = oceanic dispersal).

Great suggestion. Thank you. We have now changed the title to: “Testing the oceanic dispersal potential of Caribbean fleshy-fruited plants”.

I am a bit puzzled over the current styling of Fig 2 and Fig 3 and their titles in the main text – should these be on separate lines to show where the figures should be inserted? Especially the line/legend for Fig 2 (L422) is puzzling, as it contains a statistical result. Please correct/revise.

This formatting was requested by the journal. “Figure captions must be inserted in the text of the manuscript, immediately following the paragraph in which the figure is first cited (read order). Do not include captions as part of the figure files themselves or submit them in a separate document.” We did have to rearrange the location of these captions as we rearranged parts of the manuscript during this revision.

Depending on journal policy, it may not be possible for you to refer to the new co-author’s work as an ‘external database’, with the reference for this being an ‘in prep’ manuscript. Best to either cite the thesis (?) this comes from, or as ‘unpubl. data'. Check with the editor which is best.

We now refer to this as an unpublished database. The manuscript is now in review, but we did not want to publish a preprint yet.

L67 – insert comma after ‘context’.

Inserted

L101 – correct to ‘specialized for’

Corrected

L122 – suggest changing ‘possess’ to ‘have’

Changed

L365 – delete ‘While’ to start sentence with ‘The largest’

Deleted

L497 – change to ‘our study’

Changed

L958 – correct to ‘Flotation’ in first word of legend.

I learned during this edit that both spellings are accepted, but “floatation” is less common. Therefore, I changed it to “flotation”.

Figure 2 – italicize the species names

Italicized.

7. PLOS authors have the option to publish the peer review history of their article ([what does this mean?](https://track.editorialmanager.com/CL0/https:%2F%2Fjournals.plos.org%2Fplosone%2Fs%2Feditorial-and-peer-review-process%23loc-peer-review-history/1/010f019b2aee5d27-2da9ab6a-a4e9-4867-bbf5-9290aaf4db8a-000000/RtbzzSCLvZ8-L5kJPbUyDyLRHlX8xchAd1cmHfvvB5c=241)). If published, this will include your full peer review and any attached files.

If you choose “no”, your identity will remain anonymous but your review may still be made public.

**Do you want your identity to be public for this peer review?** For information about this choice, including consent withdrawal, please see our [Privacy Policy](https://track.editorialmanager.com/CL0/https:%2F%2Fwww.plos.org%2Fprivacy-policy/1/010f019b2aee5d27-2da9ab6a-a4e9-4867-bbf5-9290aaf4db8a-000000/sc89lQS4aDm4Id5HM-rACULCAeCy0LUihr_bQhQkHVY=241).

Reviewer #1: No

Reviewer #2: **Yes:** Yurena Arjona

Reviewer #3: No

[NOTE: If reviewer comments were submitted as an attachment file, they will be attached to this email and accessible via the submission site. Please log into your account, locate the manuscript record, and check for the action link "View Attachments". If this link does not appear, there are no attachment files.]

To ensure your figures meet our technical requirements, please review our figure guidelines: <https://journals.plos.org/plosone/s/figures>

You may also use PLOS’s free figure tool, NAAS, to help you prepare publication quality figures: <https://journals.plos.org/plosone/s/figures#loc-tools-for-figure-preparation>.

NAAS will assess whether your figures meet our technical requirements by comparing each figure against our figure specifications.
