# Supplementary material for: Testing the oceanic dispersal potential of Caribbean fleshy-fruited plants
Source: PLoS One. 2026 May 27;21(5):e0348628. doi: 10.1371/journal.pone.0348628 (PMC13215481; doi:10.1371/journal.pone.0348628)
Supplement: Attachment — Submitted filename: Response_to_Reviewers_auresp_3.docx [file pone.0348628.s016.docx]

Seokmin Kim

Reviewer Responses

PLOS One

April 15, 2026

Reviewer #1: This is my third review of this manuscript, and already on my previous review I identified no relevant concerns. The minor issued raised on my second review have now been properly solved, and I'm satisfied with the changes made.

Thank you for your comments and support. We appreciate you helping improve this manuscript.

Reviewer #2: The revised version of the manuscript, now entitled "Testing the oceanic dispersal potential of Caribbean fleshy-fruited plants" by Kim et al., has notably improved through the revision process. I appreciate the authors' effort in addressing the reviewer's concerns. This new version of the manuscript is clearer and better structured, and the use of the bibliography is also more coherent.

Thank you for your comments and support. We appreciate you helping improve this manuscript.

I only have some minor comments:

L. 68: It could be rephrased like "Another mechanism potentially involved in LDD of fleshy fruited plants" to avoid the use here of "utilize".

Change made.

L. 87: Please take care of the use of punctuation here. The reference "[27]" should come before the comma as it refers to "wind dispersal". After the reference, the break is over, so mark it with another dash: "such as anemochory — wind dispersal [27] — and epizoochory — attachment to feathers".

Change made.

L. 95: Do those large-fruited plants have always fleshy fruits? A clarification here might be good to link the previous and the next paragraphs.

Clarified to “large, fleshy-fruited plants”

L. 106-112: Include the assessment of the effect of human usage in the specific objectives and predictions.

Added “(4) Can human usage serve as an alternative predictor of species distributions?” in line 108-109.

L. 175: 10% of the floating fruits, right?

Correct. Now reads: “To conduct the tetrazolium test, we arbitrarily removed 10% of the floating fruits per species…”

L. 193-194: Based on Table 1, the two response variables used here are counts (number of days), for this kind of data a generalized linear mixed model fitted with a Poisson family is more adequate.

Clarified it to: “We then conducted generalized linear mixed model regressions fitted with a Poisson family…”

L. 308: 6% "of the fruits"

Corrected.

L. 308-310: I would place this sentence at the end of the paragraph, after discussing all flotability and viability results per species.

Moved.

L. 361: "Utilize" sounds a bit awkward in this context, as it evokes an intended decision.

Changed to “these plants can also benefit from”

L. 403: Should it not be "density and size" instead of "buoyancy"? Fruit density and size were the fruit characteristics assessed here in relation to species flotability.

We think buoyancy is more appropriate here as that was what we were ultimately aiming to evaluate. We mention density and size as the characteristics that we assessed in the previous sentence.

L. 508: The doi corresponds to the supplementary material of the paper.

Fixed.
